# Supplementary material for: Reviewing the Review: A Pilot Study of the Ethical Review Process of Animal Research in Sweden
Source: Animals (Basel). 2021 Mar 5;11(3):708. doi: 10.3390/ani11030708 (PMC8002130; doi:10.3390/ani11030708)
Supplement: Supplementary file 1 [file animals-11-00708-s001.zip › animals-1132738/Document S1- Appendix II-guidelines for data analysis.pdf]

# Appendix II

## Guide for analysis of applications and decisions of ethical approval

<u>General aspects to consider:</u>
To distinguish between sufficient and insufficient/unclear information or for example reasoning, description and motivation, be sure to focus on what is asked of the applicant or AEC. That is, how the regulation is formulated. An answer which replies to the question *if* there are negative consequences for the animals does not necessarily answer the question of *how* these consequences affect the animals etc.

Y = Yes
I = Incomplete/Insufficient/Indeterminable
N = No

*If pages are missing?*

- Specify what is missing in the cell right below the ID-number of the documents. Write **?** in the cells for which demands could not be assessed due to this.

**<u>Guidelines for assessing the applications:</u>**

*"When planning a project, the following should be considered, motivated and described…":*

**Scientific end-point(s)**
Y = Clear time reference and/or description of evaluation points related to the animals or the results of the study to be reached for the animal use within the project to be concluded.
I = Scientific end-point is described (and thereby considered) but only on a superficial level without clear time reference or evaluation points.
N = None of the above is fulfilled or if humane end-point is incorrectly described instead.

**Humane end-point(s)**
Y = An assessment template for evaluating the animals health, behaviour, pain or other indicators of reduced welfare (for example from Karolinska Institutet or Uppsala University) is specified for use and provided as an appendix to the application, or clear evaluation criteria are otherwise specified and described.
I = An assessment template is specified but not included in the application or only unclear/broad evaluation criteria such as "decreased mentation", "manifestation of side effects" etc. are described.
N = None of the above is fulfilled or if scientific end-point is incorrectly described instead.

**Clear evaluation criteria for humane end-point(s)**
Y = The evaluation criteria provided are well suited for the procedure(s) which the animals are subjected to and for what could be expected to arise as a result of or in relation to these. The criteria are to be relevant for the part of the project for which they are given.
I = Criteria are only described in general terms as for example: "complications" or "manifestation of side effects". Also if an assessment template is specified but not included.
N = None of the above is fulfilled or if scientific end-point is incorrectly described instead.

**The need for monitoring**
*Please note:* Legislation specifies that it is monitoring which is to be described and motivated and not for example handling of the animals. Be sure to distinguish between these!

Y = The need of monitoring specifically related to the experiment/procedure and its potential effect on the animal. Daily monitoring such as cage cleaning and feeding are not included. It is however enough that the application for example notes *that* extra attention will be provided after a procedure. It is the connection between the monitoring and the procedure which is of interest for this criteria. *What* the monitoring constitutes of or *how* it is structured is <u>not</u> to be included in the judgement.
I = Daily monitoring not linked to the procedure(s) is described.
N = Monitoring related to the experiment/procedure is not described.

**How the animals' pain, discomfort or other suffering is determined**
*Please note:* This includes the animals' wellbeing/suffering throughout the project and not just concerning end-points.

Y = An assessment template for evaluating the animals' pain, discomfort or other suffering (for example from Karolinska Institutet or Uppsala University) is specified for use and provided as an appendix to the

application, or clear evaluation criteria are otherwise specified and described. *How* the animals' suffering is to be assessed is described, not just *that* suffering is expected to happen.
I = An assessment template is specified but not included in the application or only unclear/broad evaluation criteria such as "decreased mentation", "manifestation of side effects" etc. are described.
N = None of the above. Suffering is perhaps mentioned but *how* it is to be assessed is not described.

*The non-technical project summary*

**Inform about the animals' suffering within the non-technical project summary**
Y = The animals' suffering is described with regards to kind of suffering, duration and/or degree of severity. Furthermore, some form of reasoning is included around how the suffering may be perceived by the animals so that laymen as well as informed researchers may appreciate the extent of the suffering.
I = The animals' suffering is briefly mentioned or described in general terms. For example "symtoms will only arise at the end of the study" or "the suffering is expected to be generally mild". No reasoning is included.
N = The animals' suffering is not mentioned.

**How the 3R (Replace, Reduce and Refine) have been fulfilled by the applicant**
*Please note:* legal requirements regarding 3R for the applicants includes an emphasis on *how* the requirements have been met, not just *that* they have been met.
*Please note further:* Only the R addressed is eligible to be judged. If one R has been inaccurately described as another, the R being described is to be judged and categorised as Y, or I. It should however be specified in the Excel sheet that said description was ascribed the wrong R.
*Please note even further:* The judgement of the 3Rs apply to whatever is specified about them within the realms of the NTPS!

**Replace**
Y = Specified *how* Replace has been applied and fulfilled in this particular project. *Why* a certain animal model has been applied in this case is described, not just that "animal models are the only available options" or "the proposed model is standard" etc. *Why* are live animals needed?
I = General explanations such as "the proposed model is the most established model", "no alternative methods exist" or "for the proposed study, live animals are needed". *That* Replace has been applied but not *how*.
N = No strategy for application of Replace is mentioned.

**Reduce**
Y = Specified how Reduce has been applied and fulfilled in this particular project. *How* the applicant has ensured the use of the least possible number of animals is described, not just *that* it has been done.
I = General explanations such as "by carefully planning the project, the number of animals needed has been kept to a minimum" or "only as many animals as are required to obtain scientifically valid results will be used" etc. *That* Reduce has been applied but not *how*.
N = No strategy for application of Reduce is mentioned.

**Refine**
Y = Specified *how* Refine has been applied and fulfilled in this particular project. Evidence of the suffering and/or vulnerability of the animals having been considered.
I = General explanations such as "educated staff makes the handling of the animals efficient and quick as possible" or "the methods used are common and reliable". *That* Refine has been applied but not *how*.
N = No strategy for application of Refine is mentioned.

**The applicant's harm-benefit analysis (HBA)**
*Please note:* There is no legal requirement that the applicant should perform a HBA when planning a project. It is however included in the Swedish application form whereby it is to be judged within the realms of this study. The application form requires a description of *"how the applicant has reasoned when reaching the conclusion that the benefit of the project exceeds that of the suffering of the animals"*.

**Harm**
Y = Requires a description of the degree of suffering caused throughout this particular project within the appointed part of the application form. It is not sufficient to simply mention the word suffering. Preferably, said harm is also weighed against the benefit although this is not required.
I = Suffering is mentioned within the appointed part of the application form but not described. General terms such as "we believe therefore that the benefit of the project surmounts the suffering of the animals".
N = Harm is not mentioned at all within the appointed part of the application form.

**Benefit**
Y = Requires a description of the expected benefit of this particular project within the appointed part of the project form. It is not sufficient to simply mention the word benefit. Preferably, said benefit is also weighed against the harm although this is not required.

I = The expected benefit of this particular project is not mentioned within the appointed part of the application form but the benefit of similar research or of research within the same field is. For example, "increased knowledge about the underlying causes of diseases is of great importance for the development of efficient drugs" or "the need for treatment is great".
N = Benefit is not mentioned at all within the appointed part of the application form.

**Guidelines for assessing the decisions:**

**How the fulfilment of 3R (Replace, Reduce and Refine) has been controlled by the AEC**
*Please note*: This has so far not been included in any of the decisions which were reviewed for practice while creating the Excel sheet, and as such no guidelines for how to assess this have been created.

**The AEC's harm-benefit analysis (HBA)**

**Harm**
Y = Requires a reasoning more elaborate than the boilerplate specified below.
I = The boilerplate "The committee considers the importance of the project to outweigh the suffering of the animals." is included in the decision. No further motivation or reasoning is given.
N = Harm is not mentioned at all within the decision.

**Benefit**
Y = Requires a reasoning more elaborate than the boilerplate specified below.
I = The boilerplate "The committee considers the importance of the project to outweigh the suffering of the animals." is included in the decision. No further motivation or reasoning is given.
N = Benefit is not mentioned at all within the decision.

**Motivation of the decision**
Y = Requires a motivation/reasoning more elaborate than the boilerplate specified below. *On what grounds* was the project approved or declined? *Why*?
I = The boilerplate "The committee considers the importance of the project to outweigh the suffering of the animals." is included in the decision. No further motivation or reasoning is given.
N = No motivation/reason behind the decision is given.

**Difference of opinion?**
Y = One or several differences of opinion have occurred and are detailed in the decision.
I = It is unclear from the available documentation if one or several differences of opinion have occurred.
N = No difference of opinion has occurred.
