# Supplementary material for: Reviewing the Review: A Pilot Study of the Ethical Review Process of Animal Research in Sweden
Source: Animals (Basel). 2021 Mar 5;11(3):708. doi: 10.3390/ani11030708 (PMC8002130; doi:10.3390/ani11030708)
Supplement: Supplementary file 1 [file animals-11-00708-s001.zip › animals-1132738/Table S1 - Appendix I-score sheet with full results .pdf]

Appendix I

Please note that original text is in Swedish. English translation (our own) is found in square brackets.

Applicable legislation **and** corresponding part of application**:**

THE APPLICANT shall provide the following information

Applicable legislation and corresponding part of application	Requirement		A	A	A	B	B	B	C	C	C	D	D	D	E	E	E	F	F	F	Totalt Y	Totalt I	Totalt N
		**Document number -->**	**a1**	**a2**	**a3**	**b1**	**b2**	**b3**	**c1**	**c2**	**c3**	**d1**	**d2**	**d3**	**e1**	**e2**	**e3**	**f1**	**f2**	**f3**	**Y**	**I**	**N**
General content requirements<br>SJVFS 2012:26 chp 3 section 4	*Vid planering av försök ska följande särskilt* ***övervägas, motiveras*** *och* ***beskrivas***<br>*[During project planning, the following should be* ***considered, motivated*** *and* ***described*** *]:*																						
2 Syfte m.m [purpose etc.]--> Beskrivivning av försökets slutpunkt [Description of the project's end point]	*1. Motivering och beskrivning av* ***slutpunkt***<br>*[1. Motivation and description of* ***end point*** *]*		Y	Y	Y	Y	Y	Y	Y	Y	Y	Y	Y	Y	Y	Y	Y	Y	I	Y	17	1	0
4.1.1.2 Svårhetsgrad och avbrytningspunkt [Degree of severity and humane end-point]	*2. Motivering och beskrivning av* ***avbrytningspunkt***<br>*[2. Motivation and description of* ***humane end point*** *]*		Y	Y	Y	I	I	Y	Y	I	I	Y	Y	Y	Y	I	N	Y	Y	Y	12	5	1
SJVFS 2012:26 chp 3 kap section 5,<br>4.1.1.2?/Saknas [Missing]	*Tydliga* ***bedömningskriterier*** *för avbrytningspunkt*<br>*[Clear* ***judgement criteria*** *for humane end point]*		Y	Y	Y	I	I	Y	I	I	Y	Y	Y	I	Y	N	N	Y	I	I	9	7	2
Saknas [Missing]	*3. Hur djurens* ***smärta, obehag eller annat lidande*** *ska bedömas*<br>*[3. How the animals'* ***pain, discomfort or other suffering*** *is to be observed and determined]*		N	N	N	N	I	I	N	N	Y	Y	Y	I	I	N	N	I	I	N	3	6	9
4.1.1.1?/Saknas [Missing]	*4. Behov av* ***tillsyn***<br>*[4. The need for* ***monitoring*** *]*		I	I	Y	N	I	Y	Y	I	Y	I	N	I	I	N	Y	N	N	N	5	7	6
**Krav enligt ansökningsmallen [Demands according to the application form template]**																							
Lagrum saknas [Applicable requirement in L150 missing]<br>Egen etisk avvägning [The applicant's own ethical deliberation]	*Egen beskrivning hur sökande har resonerat när de har kommit fram till att nyttan med försöket överväger lidandet för djuren (HBA)*<br>*[The applicant's own description of how they have reasoned when coming to the conclusion that the benefit of the project outweights the harm (HBA)].*	Skada [Harm]	N	N	I	Y	I	I	N	I	N	N	Y	N	I	Y	Y	Y	Y	Y	7	5	6
		Nytta [Benefit]	I	I	Y	Y	Y	I	I	I	Y	N	Y	N	N	Y	Y	Y	I	Y	9	6	3
**Krav på populärvetenskapliga sammanfattningens innehåll [Demands on content of the NTPS (Non-Technical Project Summary)]**	*Den populärvetenskapliga sammanfattningen ska:*<br>*[The NTPS (Non-Technical Project Summary) shall]:*																						
SJVFS 2012:26 chp 2 kap section 10,<br>Beskrivning av syftet med försöket [Description of purpose/aim of the project] , Beskriving av nyttan med försöket [Description of benefit of the project]	*1. Informera om* ***försökets syfte och nytta***<br>*[1. Inform about* ***the project's purpose/aim and benefit*** *]*	**Syfte [Purpose/aim]**	Y	Y	Y	Y	Y	Y	Y	Y	Y	N	Y	N	Y	Y	Y	Y	Y	Y	16	0	2
		**Nytta [Benefit]**	I	I	Y	I	I	I	I	I	Y	N	Y	N	Y	Y	Y	Y	Y	Y	9	7	2
Beskrivning av vilken påverkan försöket förväntas ha på djuren [Description of what affect/impact the project is expected to have on the animals]	*2. Informera om* ***djurens lidande***<br>*[2. Inform about* ***the suffering of the animals*** *]*		I	N	Y	I	N	I	Y	I	I	N	Y	N	I	I	Y	Y	Y	Y	7	7	4
Använda djurarter [Species used]	*3. Innehålla information om* ***antal och typ av djur*** *som ska användas*<br>*[3. Contain information about* ***the number and type of animals*** *to be used]*		Y	Y	Y	Y	Y	Y	Y	Y	Y	N	Y	N	Y	Y	Y	Y	Y	Y	16	0	2
3R - Ersätta (Replace), 3R - Begränsa (Reduce), 3R - Förfina (Refine)	*4. Visa hur kraven på* ***ersättning, begränsning och förfining*** *i 19 § djurskyddslagen (1988:534) uppfylls (3R)*<br>*[4. Show how the requirements of* ***Replacement, Reduction*** *and* ***Refinement*** *in section 19 of The Animal Welfare Act (1988:534) are fulfilled (3R)].*	**Ersättning [Replace]**	Y	Y	I	I	I	I	Y	Y	I	N	Y	N	Y	I	Y	Y	I	Y	9	7	2
		**Minskning/Begränsning [Reduce]**	Y	I	Y	I	Y	I	I	I	Y	N	Y	N	Y	Y	I	N	I	I	7	8	3
		**Förfining [Refine]**	Y	N	Y	I	I	I	N	I	Y	N	Y	N	N	Y	Y	Y	I	I	7	6	5

Applicable legislation **and** corresponding part of application**:**

THE COMMITTEE'S tasks

Applicable legislation and corresponding part of application	Requirement		A	A	A	B	B	B	C	C	C	D	D	D	E	E	E	F	F	F	Totalt Y	Totalt I	Totalt N
		**Document number -->**	**a1**	**a2**	**a3**	**b1**	**b2**	**b3**	**c1**	**c2**	**c3**	**d1**	**d2**	**d3**	**e1**	**e2**	**e3**	**f1**	**f2**	**f3**	**Y**	**I**	**N**
General content requirements<br>SJVFS 2012:26 chp 7 section 18	*Av beslut ska följande framgå:*<br>*[The following should be decipherable from the AEC's decision:]*																						
Villkor för beslutet [Terms of approval]	*1.* ***försöksledare***<br>*[1. the* ***principal investigator*** *]*		Y	Y	Y	Y	Y	Y	Y	N	N	Y	Y	Y	Y	Y	Y	Y	Y	Y	16	0	2
Villkor för beslutet [Terms of approval]	*2.* ***föreståndare***<br>*[2. the* ***director*** *]*		Y	Y	Y	Y	Y	Y	Y	N	N	Y	Y	Y	Y	Y	Y	Y	Y	Y	16	0	2
Villkor för beslutet [Terms of approval]	*3. den* ***anläggning eller plats*** *där försöket ska genomföras*<br>*[3. the* ***location*** *where the project is to take place]*		Y	Y	Y	Y	Y	Y	N	N	N	Y	Y	Y	Y	Y	Y	Y	Y	Y	15	0	3
Nämndens beslut [The committee's decision]/ Villkor för beslutet [Terms of approval]	*4.* ***eventuella särskilda villkor*** *, inklusive om försöket ska utvärderas i efterhand och i så fall när*<br>*[4.* ***changes or additions needed*** *for approval, including whether or not the project should undergo retrospective assessment]*		Y	Y	Y	Y	Y	Y	Y	Y	Y	Y	Y	Y	Y	Y	Y	Y	Y	Y	18	0	0
Avvikande mening (--> bilaga till beslutet) [Differences of opinion]	*5.* ***eventuell avvikande mening*** *enligt 19 § Förvaltningslagen (1986:223)*<br>*[5. any* ***differences of opinion*** *according to section 19 of The Administrative Procedure Act 1986:223]*		N	N	N	I	I	I	N	N	N	N	N	N	N	N	N	N	N	Y	1	3	14
SJVFS 2012:26 chp 7 section 25,<br>Nämndens beslut / Nämndens bedömning [The committee's decision/ The committee's evaluation]	*Nämnden ska fastställa försökets* ***svårhetsgrad*** *enligt 21 § Djurskyddslagen 1988:534*<br>*[The Committee shall determine the project's* ***degree of severity*** *according to section 21 of The Animal Welfare Act 1988:534]*		Y	Y	Y	Y	Y	Y	Y	Y	Y	Y	Y	Y	Y	Y	Y	Y	Y	Y	18	0	0

SJVFS 2012:26 chp 7 section 25, 27,<br>Nämndens bedömning [The committee's evaluation]	*... den regionala djurförsöksetiska nämnden ska pröva om förutsättningarna i 19 § 1-3 Djurskyddslagen 1988:534 är uppfyllda. (**3R**)*<br>*[...the regional committee shall determine whether or not the requirements listed in Thee Animal Welfare Act 1988:534 section 19, points 1-3 have been fulfilled. (**3R**)]*<br>*/*<br>*...vid bedömningen av försöket ta hänsyn till sakkunskap särskilt inom 1. för försöket relevanta vetensakpliga områden, inkklusive frågor om ersättning, begränsning och förfining. (**3R**)*<br>*[...when reviewing the project, shall pay attention to expertise especially within 1. areas relevant for the main scientific aspects of the proposed project, including questions concerning replacement, reduction and refinement. (**3R**)]*<br>*/*<br>*...ska säkerställa att försöket är utformat så att det kan utföras på ett så skonsamt och miljövänligt sätt som möjligt (**Refine**)*<br>*[...shall ensure that the project is designed so that it can be performed in a as gentle and environmentally friendly way as possible (**Refine**)].*	**Ersättning [Replace]**	N	N	N	N	N	N	N	N	N	N	N	N	N	N	N	N	N	N	**0**	**0**	**18**
		**Minskning/Begränsning [Reduce]**	N	N	N	N	N	N	N	N	N	N	N	N	N	N	N	N	N	N	**0**	**0**	**18**
		**Förfining [Refine]**	N	N	N	N	N	N	N	N	N	N	N	N	N	N	N	N	N	N	**0**	**0**	**18**
SJVFS 2012:26 chp 7 section 25, 26, The Animal Welfare Act 1988:532 section 21,<br>Nämndens bedömning [The committee's evaluation]	*Nämnden ska dessutom göra en lidande- och nyttoanalys av försöket från etisk synpunkt där djurets lidande ska vägas mot den förväntade nytta som försöket kan komma att resultera i för människor, djur eller miljö.(**HBA**)*<br>*[The committee shall also perform a HBA of the project from an ethical point of view where the suffering of the animals is weighed against the expected benefit that the project might hold for humans, anuimals or the environment. (**HBA**)]*<br>*/*<br>*Projektbedömningen ska säkerställa att: a) projektet är motiverat från vetenskaplig synpunkt eller utbildningssynpunkt, eller lagstadgat, b) projektets syften motiverar användning av djur. (**HBA**)*<br>*[The project evaluation shall ensure that: a) the project is motivated from a scientific or educational point of view, or required by law, b) the purposes/aims of the project motivate animal use. (**HBA**)]*<br>*/*<br>*Vid prövningen av ett sådant ärende ska försökets betydelse vägas mot lidandet för djuret. (**HBA**)*<br>*[During the review of such a matter, the necessity of the project shall be weighed against the suffering of the animal. (**HBA**)].*	**Skada [Harm]**	I	I	I	N	N	N	I	I	I	N	I	I	I	Y	I	Y	Y	Y	**4**	**10**	**4**
		**Nytta [Benefit]**	I	I	I	N	N	N	I	I	I	N	I	I	I	Y	I	Y	Y	Y	**4**	**10**	**4**
**Krav på populärvetenskapliga sammanfattningens innehåll [Requirements regarding content of the NTPS]**																							
SJVFS 2012:26 chp 7 section 17,<br>Nämndens beslut - Populärvetenskaplig sammanfattning (separat mall) [The AEC's Decision –NTPS] (separate template)	*Nämnden ska **komplettera** de populärvetenskapliga sammanfattningarna med:*<br>*[The committee sha ll **amend** the NTPS with:]*																						
	*1. försökets **svårhetsgrad***<br>*[1. The **degree of severity** of the project]*		Y	Y	Y	N	N	N	N	N	N	N	I	N	N	N	N	N	N	Y	**4**	**0**	**14**
	*2. eventuella **tillägg eller ändringar** som har beslutats*<br>*[2. any **changes or additions** that the committee has decided are required]*		Y	Y	Y	N	N	N	N	N	N	N	N	N	N	N	N	N	N	Y	**4**	**0**	**14**
	*3. eventuellt beslut om **utvä rdering i efterhand** , inklusive vilka delar och ur vilken aspekt*<br>*[3. if the project should undergo **retrospective assessment** , including, if so, what parts of the project and from which aspect]*		Y	Y	Y	N	N	N	N	N	N	N	N	N	N	N	N	N	N	Y	**4**	**0**	**14**
**Motivering av beslut [Motivation of decision]**																							
SJVFS 2012:26 chp 7 section 8,<br>Beredningsgruppens förslag till nämndens ställningstagande (separat mall) [Proposition for AEC decision by the review panel] (separate template)	*Beredningsgruppen ska ta fram **skriftligt och motiverat förslag till beslut.** Om det är mer lämpligt får dock beredningsgruppen istället ta fram ett **skriftligt beslutsunderlag** där gruppen så långt det är möjligt har förberett ärendet inför nämndens sammanträde.*<br>*[The review panel shall to produce **a written and motivated proposition** . If more appropriate, the review panel may instead produce **a written decision basis** where they, to the outmost possible extent, have prepared the matter for the AECs meeting]*	**Skriftligt [Written]**	Y	Y	N	Y	Y	Y	Y	Y	N	Y	Y	Y	Y	Y	Y	Y	Y	Y	**16**	**0**	**2**
		**Motiverat [Motivated]**	N	N	N	N	N	N	N	N	N	Y	I	Y	Y	Y	Y	Y	Y	Y	**8**	**1**	**9**
The Administrative Procedure Act 1986:223 section 20,<br>Nämndens beslut / Nämndens bedömning [The committee's decision/ The committee's judgement]	***Ett beslut varigenom en myndighet avgör ett ärende skall innehålla de skäl som har bestämt utgången** , om ärendet avser myndighetsutövning mot någon enskild.Skälen får dock utelämnas helt eller delvis 1. om beslutet inte går någon part emot eller om det av någon annan anledning är uppenbart obehövligt att upplysa om skälen.*<br>*[**A decision that determines a matter should contain the reason s behind the outcome** , if the matter concerns an authority's actions against an individual. The reasons may however be left out, partially or completely, 1. if the decision does not affect a person's situation in a way that is not insignificant, or if it is for some other reason without a doubt unnecessary to inform about the reasoning behind the decision.]*		I	I	I	N	N	N	I	I	I	I	I	I	I	Y	I	Y	Y	Y	**4**	**11**	**3**
